# Rare Findings in Cleidocranial Dysplasia Caused by RUNX Mutation

**DOI:** 10.1055/s-0041-1736482

**Published:** 2021-10-22

**Authors:** Aysel Kalayci Yigin, Mehmet Bugrahan Duz, Mehmet Seven

**Affiliations:** 1 Department of Medical Genetics, Istanbul University-Cerrahpaşa, Cerrahpaşa Medical School, Istanbul, Turkey; 2 Department of Medical Genetics, Health Sciences University, Haseki Training and Research Hospital, Istanbul, Turkey

**Keywords:** cleidocranial dysplasia, RUNX mutation, Sanger sequencing, rare findings

## Abstract

**Background** Cleidocranial dysplasia (CCD, #MIM119600) is an autosomal-dominant skeletal dysplasia characterized by delayed closure of the cranial sutures, aplasia, or hypoplasia of the clavicles and dental abnormalities. These findings were accompanied by mobile and drooping shoulders, frontal and parietal bossing, hypertelorism, brachycephaly, short stature, supernumerary, and late erupting teeth. Radiographic studies can reveal involvement of multiple bones including skull, chest, pelvis, and limbs. CCD can be diagnosed with clinical and radiological evaluation and validated by molecular studies. Heterozygous loss of function *RUNX2* gene, which plays an important role in osteogenesis and differentiation of precursor cells, causes CCD phenotype.

**Methods** In this article, we reported five cases from three unrelated families with CCD phenotype. All exons and exonic–intronic boundary regions of *RUNX2* gene from five patients were analyzed by polymerase chain reaction amplification and direct Sanger-sequencing.

**Results** Our patients had classical CCD phenotype and we detected three different previously described mutations including c.1171C > T, IVS4 + 4delAAGT and c.676G > A. However, nail dysplasia has never been associated with these mutations. Our patients had varying degrees of nail dysplasia. Two of three mutations are related with Runt DNA-binding domain of *RUNX2* protein in Wnt signaling and c.1171C > T had effect on proline/serine/threonine-rich (PST) domain. Recently, Wnt signaling pathway was presented as a key regulator of digit and nail differentiation. Our data suggest that *RUNX2* gene may have an essential role on embryogenesis of nails, probably by protecting their integrity.

## Introduction

Cleidocranial dysplasia (CCD, #MIM119600) is a rare autosomal-dominant skeletal dysplasia (prevalence 1/1,000,000), with higher rates of several ethnic groups. 1 2 CCD is characterized by hypoplastic or aplastic clavicles, multiple dental abnormalities, enlarged calvaria with frontal bossing, open fontanelles, and short stature. 3 The patients with CCD phenotype have mobile and drooping shoulders that are an evidence to aplasia or hypoplasia of the clavicles. 4 Radiographic studies also reveal involvement of multiple bones including skull, chest, pelvis, vertebrae, and limbs. In addition, those findings can be accompanied by dental abnormalities including delayed eruption of secondary dentition, failure to shed the primary teeth, and supernumerary teeth with dental crowding. 5

*RUNX2* gene, located on chromosome 6p21.1, has a special role in osteogenesis and differentiation of precursor cells, and regulates chondrocyte differentiation toward hypertrophy, cell migration, and vascular invasion of bone. Osteogenesis is a process beginning from mesenchymal cells to final maturation of functional osteoblasts. In particular, *RUNX2* gene plays a role in conversion of mesenchymal cells to preosteoblastic cells. This process is followed by a transformation from preosteoblasts to functional osteoblasts, regulated by Osterix (OSX). If there is a deletion in OSX, preosteoblasts transform to chondroblasts, which are shown experimentally in mouse models. The authors also indicated that loss of function in *RUNX2* gene causes CCD, while OSX mutations give rise to a form of osteogenesis imperfecta. 6 Roles of Wnt signaling have been identified in many aspects of development. Impairment of the Wnt signaling causes several human diseases and structural deficits. 7 During skeletal development, Wnt canonical pathway regulates expression levels of *RUNX2* gene to provide osteoblastic differentiation in mouse embryonic fibroblasts, pluripotent mesenchymal, and osteoprogenitor cells in vitro. 8 Takeo et al suggest that Wnt signaling in the nail epithelium acts dual functions to promote both nail regeneration and Runx2+ mesenchymal cell growth. 9

Three different previously reported mutations in *RUNX2* gene were detected by Sanger sequencing. Most of the *RUNX2* mutations occur in individuals affected by classical CCD, although spectrum of different mutations has not been found to show nail dysplasia. In this study, five cases from three unrelated families with variable clinical spectrums in toenails with a mutation in the *RUNX2* gene are presented in Turkish population.

## Materials and Methods

### Study Population

Five cases from three unrelated families with CCD were included in the study. After full physical examination, written informed consent was obtained from each case.

### Sanger Sequencing of RUNX2 Gene

Genomic DNA was extracted from peripheral blood using standard procedures. Primers of *RUNX2* gene were designed using primer 3 software, version 4.0.0 ( http://bioinfo.ut.ee/primer3/ ). All exons and exonic–intronic boundary regions of *RUNX2* gene from 5 patients were analyzed by polymerase chain reaction amplification and direct Sanger sequencing. Sequences were compared with the wild-type sequence as submitted to Ensembl Accession number ENST00000371438.

## Results

### Clinical Investigations

#### Case Series

##### Patient 1

A 25-year-old female patient was referred to our clinic due to skeletal and dental abnormalities. She was the first child of nonconsanguineous parents. Her prenatal term was uneventful and she was born by spontaneous normal vaginal delivery at full term with normal birth parameters. Her motor-cognitive developmental milestones were in normal ranges. It was the first time; her family had realized that the primary teeth eruption was delayed compared with her peers. When she was 14 years old, all of her primary teeth were replaced with dentures by dental surgeon. Skeletal abnormalities accompanied. She was diagnosed with scoliosis and genu varum at the age of 25. She was operated for the reconstruction of these deformities within 2 years. Physical examination revealed a short stature and microcephaly. She had a body height of 142 cm (<3rd centile) and a body weight of 53 kg (10–25th centile). The head circumference was 51.5 cm (<3rd centile).

**Fig. 1:**
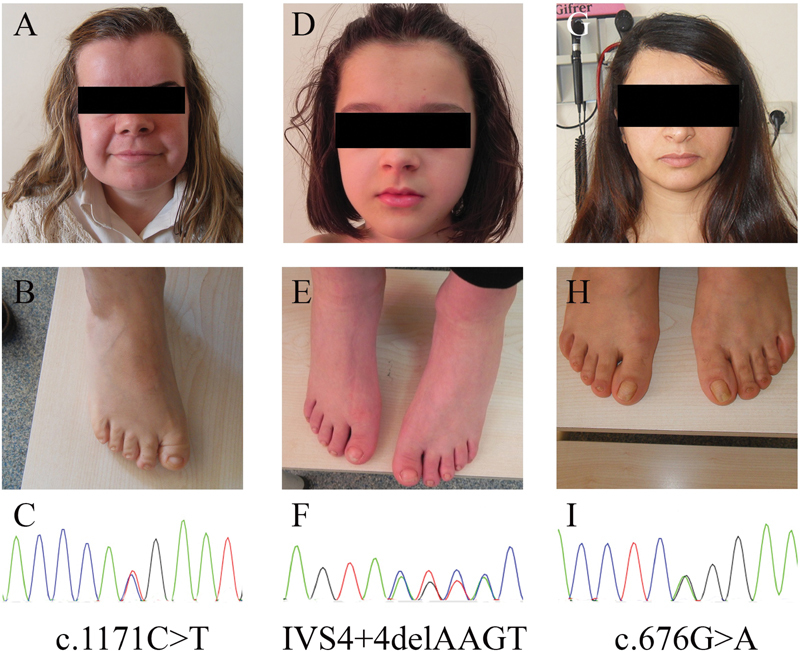
(A–I) The patient 1 to 3 facial appearance, feet, and DNA mutation.

**Fig. 2:**
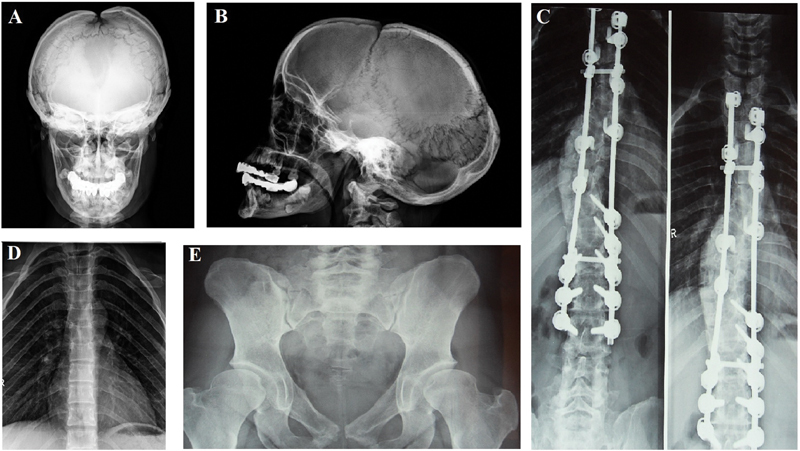
X-ray findings of the patients: ( **A** – **B** ) wormian bones, wide open anterior fontanelle, supernumerary teeth along with dental crowding; ( **C** ) scoliosis and fixation; ( **D** ) hypoplastic clavicles, short ribs, narrow thorax; and ( **E** ) wide pubic symphysis, broad femoral head, hypoplastic iliac wing.

Several dysmorphic findings were noted as prominent forehead, metopic depression, bitemporal narrowing, hypertelorism, down-slanting palpebral fissures, wide nasal tip, depressed nasal bridge, and midface retrusion. Other systemic evaluation revealed wide-open anterior fontanel; narrow thorax; pectus excavatum; narrow sloping shoulders that opposed at the midline; scoliosis; brachydactyly; small hands with long middle finger; short, broad, and outward bending thumb; and clubbing of toes ( Fig. 1 ). Toenails were dysplastic, located abnormally and originated from the distal of the finger. Radiography demonstrated that delayed fontanel closure, clavicular hypoplasia, narrow upper thoracic diameter, scoliosis, and supernumerary teeth along with dental crowding ( Fig. 2 ). Osteopenia was also detected by bone densitometry and she was given treatment.

Her father and grandmother also had similar clinical findings including prominent forehead, metopic depression, 2 × 3 cm fibrous tissue covered anterior fontanel, hypertelorism, down-slanting palpebral fissures, and midface retrusion. The thumb is thick and deviated outside, and the second finger is long. He had dysplastic nails, small feet, and clubbing of toes. The father had similar radiological findings with her daughter. He had dentures like her daughter due to failure to shedding primary teeth.

##### Patient 2

An 11-year-old female patient referred to our center with backache and scoliosis. She was the fourth child of nonconsanguineous parents. There was no other individual with similar complaints in her family. Her full-term prenatal life was uneventful with a normal spontaneous vaginal delivery. She had normal birth parameters. She had normal neurodevelopmental milestones. Her family had noticed that she had a wide anterior fontanel and scoliosis when she was 5 years old. At the time of admission, she had short stature and microcephaly. She had a body height of 130 cm (<3rd centile) and a body weight of 27 kg (3–10th centile). The head circumference was 52.5 cm (50th centile).

She had several clinical dysmorphic features including brachycephaly, prominent forehead, marked and bow shaped eyebrows, hypertelorism, telecanthus, small nose with wide nasal root, upturned nostrils, midface retrusion, narrow and high palate, prognathism, deficient and irregular and notched primary teeth, brachydactyly and outward deviated thumb, small feet, dysplastic toenails, and outward deviated broad big toe ( Fig. 1 ). Her fontanels were closed. She had joint laxity, pectus excavatum, narrow sloping shoulders that opposed at the midline, short thorax, long arms with curved forearms and scoliosis ( Fig. 2 ). She was operated two times for scoliosis. Radiographic investigation determined brachydactyly, clavicular hypoplasia, narrow thorax, and scoliosis. Clinical and radiological evaluations were normal in her parents.

##### Patient 3

The proband was consulted to our department for her supernumerary teeth when she was 30 years old. There was no information about her birth parameters and details; however, it was known that she had good neurological development. Her primary teeth erupted on time compared with her peers. When she was 7 years old, her primary teeth had changed to dysplastic permanent teeth. These teeth decayed 2 years later and nine of these were replaced with dentures. As expected in this condition, dental eruption was still in progress and X-rays of the jaw revealed six unerupted teeth. On her examination, the patient had short stature with 152 cm height (3th centile); her weight was 48 kg (3–10th centile) and head circumference was 52.5 cm (50th centile).

**Fig. 3:**
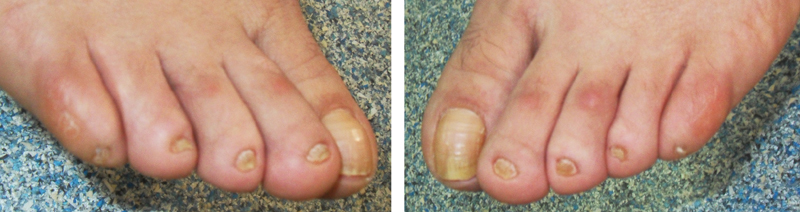
Nail hypoplasia of the third patient.

On physical examination, we noted frontal and occipital bossing, brachycephaly, open anterior fontanelle, low nasal bridge, hypertelorism, midface hypoplasia, irregular teeth, micrognathia, mobile and drooping shoulders, narrow thorax, small scapulae, small hands and thin, long fingers and clubbed toes ( Fig. 1 ). Interestingly, bilateral nail hypoplasia of toes, which is an unusual finding for CCD, was found during extremity inspection ( Fig. 3 ). Radiographic studies were suggested hypoplastic clavicles, Wormian bones, bossing of frontal and occipital bones, calvarial thickening, wide symphysis pubis, broad femoral head with short femoral neck, hypoplastic iliac wings, and supernumerary teeth along with dental crowding ( Fig. 2 ). Her parents could not be examined in terms of skeletal dysplasia, because they were deceased. In her family history, nobody had neither short stature nor similar nail findings. The clinical information of all patients is given in Table 1 .

**Table 1:** Features of three families with cleidocranial dysplasia

	Family 1			Family 2	Family 3
	Proband	Father	Grandmother		
Age	26	58	70	14	30
Gender	Female	Male	Female	Female	Female
Short stature	+	+	+	+	+
Brachycephaly	+	+	−	+	+
Delayed closure of fontanels	+	+	+	+	+
Frontal bossing		+	+	+	+
Parietal bossing	+	−	−	−	−
Wormian bone	+	−	−	−	+
Hypertelorism	+	+	−	+	+
Hypoplasia of the maxilla	+	+	+	+	+
Supernumerary teeth	−	−	−	−	+
Delayed eruption of secondary dentition	+	+	+	+	+
Clavicular hypoplasia	+	+	+	+	+
Mobility of droopy shoulders	+	+		+	+
Wide pubic symphysis	−	−	−	−	+
Vertebral alteration(scoliosis)	Severe	Mild	Mild	Severe	−
Brachydactyly	+	−	+	−	−
Hypothyroidism	+	−	−	−	−
Hearing loss	mixed type	Mild sensorineural	−	−	−
Family history	Familial			De novo	N/A
Nucleotide change	c.1171 C > T			IVS4 + 4delAAGT	c.676G > A
Mutation		R391X		−	R225Q

## Discussion

CCD (#MIM119600) is an autosomal-dominant skeletal dysplasia characterized by delayed closure of the cranial sutures, aplasia or hypoplasia of the clavicles, and dental abnormalities. 10

In the first family, the proposita had classical CCD phenotype, accompanied by hypothyroidism and mixed type hearing loss. Mutation analysis of this family revealed c.1171C > T, p.R391X in *RUNX2* gene. Her father with the same mutation had no evidence to suggest skeletal dysplasia except short stature, while he had dental complaints including late erupting secondary dentition, facial dysmorphism, and a mild sensorineural deafness which is a rare audiological finding in CCD patients. The proband had mixed type hearing loss. In a study, incidence of mixed type hearing loss in CCD was reported 33%. 11

RUNX protein contains a highly conserved QA, Runt, and PST domains, which are rich in the amino acids. c.1171C > T mutation may have effects on PST domain which has a crucial role in both transactivation and transcription repression. 12 Our second case with heterozygous splice site IVS4 + 4delAAGT mutation on runt domain may consequently affect this functional part of the protein. Although she had severe dental abnormalities, she had a milder CCD phenotype than the other two patients. Evaluating her family results indicated that the proposita of the second family had de novo mutation. IVS4 + 4delAAGT mutation was reported previously, but her clinical findings were missing. 13 To the best of our knowledge, this is the first case with mutation analysis and clinical findings.

The family of the third case had heterozygous missense c.674G > A (p.R225Q) mutation causing to change arginine residue with glutamine at the 225th position of RUNX2 protein. Since family members could not be reached, it is not determined whether the mutation is de novo or not. This R225Q mutation causes dental and skeletal problems, reported several times previously. 14 15 16

Previously reported three mutations in *RUNX2* gene were detected by Sanger sequencing. Most of the *RUNX2* mutations occur in individuals affected by classical CCD. Nail dysplasia was not seen in carriers of these mutations in classical CCD. Tang et al reported a large family with CCD phenotype and molecular analysis revealed heterozygous c.407T > C mutation in *RUNX2* . Interestingly, all patients with mutation had hyperplastic fingernails. 17 However, our cases had different mutations than c.407T > C mutation and variable degrees of dysplastic toenails. The phenotypic differentiation of nail dysplasia in our CCD patients with an autosomal-dominant inheritance pattern may have been caused by either poor penetrance or variability of expression. In addition, two of three mutations are related to Runt DNA-binding domain of RUNX2 protein and c.1171C > T had effect on PST domain. In canonical Wnt signaling pathway, activation of Wnt promotes nail regeneration and Runx2+ related mesenchymal cell growth. 9 It is well-known that Runx2 is targeted by Wnt signaling for the early specification of the osteoblast lineage. Besides, Gaur T et al considered that canonical WNT signaling directly regulates *RUNX2* gene expression in early skeletal development. 8 Taken together, these findings suggested that WNT signaling and *RUNX2* gene highly interact with each other. WNT signaling promotes nail regeneration; therefore, disease causing mutations may lead to nail hypoplasia in these patients. Although many Runt domain-related mutations have been reported for classical CCD phenotype in the literature, the possible association with nail abnormality is second reported here. 17

It has been difficult to demonstrate genotype–phenotype correlation for *RUNX2* because of very variable phenotypic penetrance of the mutations. Majority of *RUNX2* variants are seen in patients with classic CCD, but nail dysplasia with variable spectrum is an unrecognized phenotype. Each case in our study had different mutations with variable degrees of nail dysplasia.

## Conclusions

In conclusion, the first report on CCD in Turkish population may be of importance to associate *RUNX2* mutations with nail morphogenesis.
